# Direct and Indirect Effects of Elementary School Stroke Education in Akashi City: Before-Class Versus After-Class Test Comparison

**DOI:** 10.7759/cureus.103746

**Published:** 2026-02-16

**Authors:** Hiroshi Hase, Koshi Nakagawa, Yuzuru Tanizawa, Masakazu Mishina, Syuuji Horiguchi, Hideharu Tanaka

**Affiliations:** 1 Emergency Medical Services, Akashi City Fire Department, Akashi, JPN; 2 Department of Integrated Science and Engineering for Sustainable Societies, Faculty of Science and Engineering, Chuo University, Tokyo, JPN; 3 Graduate School, Emergency Medical System, Kokushikan University, Tama, JPN

**Keywords:** elementary school student, emergency medical technician, hemiplegia, intergenerational knowledge transfer, stroke education, stroke prevention, stroke recognition ability, stroke symptoms awareness

## Abstract

Objective: This study examined whether elementary school students could acquire knowledge about stroke symptoms and responses by attending a class taught by an emergency medical technician. This study determined if students can communicate the same content to their guardians.

Methods: Between September 2014 and March 2019, emergency medical technicians conducted stroke education classes for elementary school students in Akashi City, Hyogo Prefecture. Students who attended the classes subsequently conveyed their knowledge to their guardians. Both the students and guardians who received information from them were included in the analysis. A nine-point stroke knowledge test was administered to students and guardians before and after the class, and the mean total scores were compared using a paired t-test.

Results: In total, 5,520 elementary school students and 4,132 guardians participated in this study. In the nine-point test administered before and after the class, the mean score among students significantly increased from 6.3 to 8.7 (difference: 2.39, 95% confidence interval (CI): 2.39-2.41, p < 0.01), and the mean score among guardians significantly increased from 7.6 to 8.5 (difference: 0.92, 95% CI: 0.91-0.93, p < 0.01).

Conclusion: After the school-based educational intervention, short-term improvements in stroke-related knowledge were observed among elementary school students, with corresponding improvements also observed among guardians.

## Introduction

In Japan, stroke-related deaths accounted for 7.3% of all deaths in 2021. Stroke remains the fourth leading cause of death in Japan, accounting for approximately 100,000 deaths annually [1]. Moreover, it is the second leading cause for long-term care [2]. In Japan’s rapidly aging society, addressing the increase in the incidence of stroke and establishing early treatment methods are urgent public health issues. The prompt treatment of stroke is crucial to a favorable prognosis among affected patients, including intravenous thrombolysis with alteplase within 4.5 hours of its onset [3].

In prehospital settings, efforts have been made to improve the education of emergency medical technicians and standardize emergency response activities aimed at improving the outcomes of stroke patients. These initiatives have led to improved stroke recognition ability by paramedics in the field [4], as well as more efficient collection of patient information, observation, treatment, and decision-making, ultimately resulting in reduced on-site activity time [5]. However, despite recognizing symptoms suggestive of stroke, some laypersons do not seek immediate medical attention and instead opt to observe the situation [6]. Therefore, it is necessary to educate the public about the importance of early detection of stroke symptoms and the need to make appropriate emergency calls to the hospital.

The necessity of stroke education for children is increasingly recognized [7], and several reports have demonstrated its effectiveness in increasing knowledge among children from kindergarten to junior high school [8-10]. For example, recent international studies, including those on the FAST Heroes educational program, have demonstrated that school-based stroke education enables children to effectively transfer stroke-related knowledge to their parents and grandparents, and that such knowledge can be retained for several years after program implementation [11-12]. These findings reaffirm the importance of providing compulsory preventive education and increasing awareness among laypersons [13-14]. Furthermore, adopting active learning methods in elementary school classes, which encourage active participation in the learning process, may enhance the acquisition and retention of stroke-related knowledge among students. By sharing this knowledge with their guardians, students may contribute to improving awareness among the broader public [15]. Therefore, stroke education, such as cardiopulmonary resuscitation (CPR) training, should be integrated into compulsory education [16-17]. The physical education guidelines, which include “disease prevention,” [18] could incorporate stroke education in the current Japanese elementary school curriculum. This would allow for continuous, rather than one-time, awareness through formal education.

Previous studies have implemented stroke education programs targeting elementary school students and their guardians [15]. However, while the transmission of knowledge from students to guardians was observed, objective evaluations using standardized indicators remain limited, and further research is needed [9]. Therefore, this study examines whether providing stroke education classes to elementary school students would enable them to acquire stroke knowledge and effectively convey this knowledge to their guardians, thereby increasing family-wide stroke awareness.

In contrast to other countries, reports of such educational programs and related research are limited in Japan [15]. Against the backdrop of a rapidly aging society, stroke education for elementary school students is expected to contribute to the effective dissemination of stroke-related knowledge and increased awareness within the broader community. Therefore, this study aimed to objectively examine whether elementary school students can acquire stroke-related knowledge through a school-based stroke education class and whether they can appropriately convey this knowledge to their guardians, using standardized tests administered before and after the class.

## Materials and methods

Study design

This study used a before-and-after design and was approved by the Ethics Committee of Kokushikan University (approval no. 23024).

Of the 28 public elementary schools in Akashi City, Hyogo Prefecture, this program was implemented in schools that expressed interest and provided consent to participate. School selection was not randomized; therefore, this study used convenience sampling based on participation in a city-wide stroke education program.

Before the study period, emergency medical technicians visited the elementary school. They provided the principal and teacher with detailed verbal and written explanations of the class content and tests and obtained their consent to proceed with the class. Following this, the guardians received detailed written information a few days prior to the study and provided written consent for their participation. Finally, the students received a thorough verbal explanation of the class content and tests on the day of the class.

Study participants

The study participants were elementary school students aged 10 to 12 years, who attended stroke education classes conducted by emergency medical technicians in Akashi City, Hyogo Prefecture, between September 1, 2014, and March 31, 2019, and their guardians. The age group of the student participants was selected on the advice of the Akashi City Board of Education, which is responsible for the local school education administration. The board indicated that students aged 10 to 12 years would understand the class content, have more opportunities to discuss it with their families, and convey the information effectively. The inclusion criteria for students were enrollment in an elementary school in Akashi City, Hyogo Prefecture; attendance at a stroke education class delivered by emergency medical technicians during the study period; and completion of both the pre-class and post-class tests. The exclusion criteria were absence on the day of the class, non-submission of the test, or invalid responses. Guardians who received stroke-related information from participating students and completed both the pre-class and post-class tests were included in the analysis.

Class content and communication to guardians

The curriculum of the stroke education class implemented in this study is shown in Table 1. The curriculum was developed collaboratively with the National Cerebral and Cardiovascular Center, based on previously reported school-based stroke education programs. Each class lasted 45 minutes and was conducted in Japanese. The instructors were emergency medical technicians affiliated with the Akashi City Fire Department, and all instructors delivered the class using a unified curriculum and teaching materials developed under the supervision of neurologists. Each emergency medical technician was responsible for approximately 30 students. The class consisted of three components: 1) an animated video about stroke; 2) a lecture on stroke symptoms, risk factors, appropriate responses upon stroke onset, and the FAST acronym (Face, Arm, Speech, and Time); and 3) a hands-on activity to help students understand hemiplegia. After the class, students were instructed to convey the class content to their guardians at home using a comic book on stroke distributed by the school. Students were not provided with any specific guidelines for this communication method or duration. The reliability and validity of the educational content were ensured through collaboration with a specialized medical institution, standardization of teaching methods, and alignment with stroke education frameworks reported in previous studies.

**Table 1:** Course curriculum implemented for elementary school students

Content	Duration	Details
Introduction	5 minutes	Introductory lecture
		1. Self-introduction
		2. About the work of the Fire Department
		3. Need for stroke education
Watching anime	10 minutes	Watch an anime about stroke: "What is stroke?"
Lecture on stroke	10 minutes	Review the anime using slides
		1. Risk factors for stroke
		2. Symptoms of stroke
		3. Response to stroke onset
		4. FAST: Face, Arm, Speech, Time–Call an Ambulance!
Experience hemiparesis	15 minutes	Experience physical impairment to understand hemiparesis
Summary	5 minutes	Summary of the lesson content

Outcome and measurement process

The primary outcome was the test score retrieved from the students before and after the educational intervention. The secondary outcome was the accuracy rate for each question in the questionnaire. The test comprised four questions based on four topics: stroke symptoms, response to stroke onset, stroke risk factors, and FAST. Each correct response was awarded one point; three correct items were awarded for Question 1, one for Question 2, four for Question 3, and one for Question 4, resulting in a total score of 9 points (Table 2).

**Table 2:** Tests administered to elementary school students and their guardians before and after classes Reproduced from Tomari S et al. [13] with permission from the original authors.

Questionnaire about stroke (cerebral infarction ・ cerebral hemorrhage)																	
Elementary school ( ____________ ) age ( ___ years) class attendance record number (no. )																	
Question 1: Which of the following symptoms do you think are most likely during a stroke? (Any number of answers.)																	
1. One side of the face droops																	
2. Farting																	
3. Stiff shoulders																	
4. Difficulty speaking																	
5. Fever																	
6. One side of the body does not move																	
7. Abdominal pain																	
Question 2: What should you do when someone may have had a stroke? (Choose only one.)																	
1. Tell them to take their own car, a bus, or a cab to the hospital.																	
2. Tell them to go to a hospital tomorrow.																	
3. Call 119 for an ambulance.																	
4. Tell them to lie down and get some rest.																	
Question 3: Which of the following are possible causes of stroke? (Any number of answers.)																	
1. Hypertension																	
2. High cholesterol																	
3. Lumbago																	
4. Alcohol consumption																	
5. Constipation																	
6. Tobacco																	
7. Millionaire																	
Question 4: When you have a stroke, what does each of the following mean: “Let’s go to the hospital because of your face・arm・speech”? (One answer for each.)																	
1. What about “Face”?																	
A. The person can smile.																	
B. One edge of the mouth is drooped or distorted.																	
C. The person cannot smile on both sides of the face.																	
2. What about “Arm”?																	
A. Lack of strength in one arm.																	
B. I can’t raise both arms.																	
C. One's arm is broken.																	
3. What about “Speech”?																	
A. The person becomes chatty.																	
B. The person has difficulty speaking.																	
C. The person uses worse language.																	

The pre-class test for students was conducted either the day before or on the day of class. The post-class test was conducted on the following day or the day after the class, after the students had conveyed the class content to their guardians at home, using the stroke comic book to evaluate knowledge retention through this transmission process.

The pre-class test for guardians was filled-in at home using test sheets shared with students several days before the class. The post-class test was completed at home within one week-after the students explained the class content to their guardians using the stroke comic book, to evaluate whether the content had been properly conveyed. The completed tests were then submitted to the school by the elementary school students.

Statistical analysis 

Descriptive statistics were used to summarize the participants’ characteristics and were reported as numbers and percentages. To analyze the test scores before and after the educational intervention, a paired t-test was performed, and the differences and 95% confidence intervals (CIs) were calculated. The distribution of score differences before and after the educational intervention was visually assessed using Q-Q plots. No substantial deviations from normality were observed, and given the large sample size of this study, the paired t-test was applied in consideration of its robustness to mild departures from normality. For the secondary evaluation items, McNemar’s test was performed for hypothesis testing to compare the correct answer rates for each question before and after the educational intervention, and kappa coefficients with 95% CIs were calculated. The significance level for all statistical analyses was set at 0.05 (two-tailed). All statistical analyses were performed using JMP Pro ver. 15 (SAS Institute, Inc., Cary, NC, USA).

## Results

**Figure 1:**
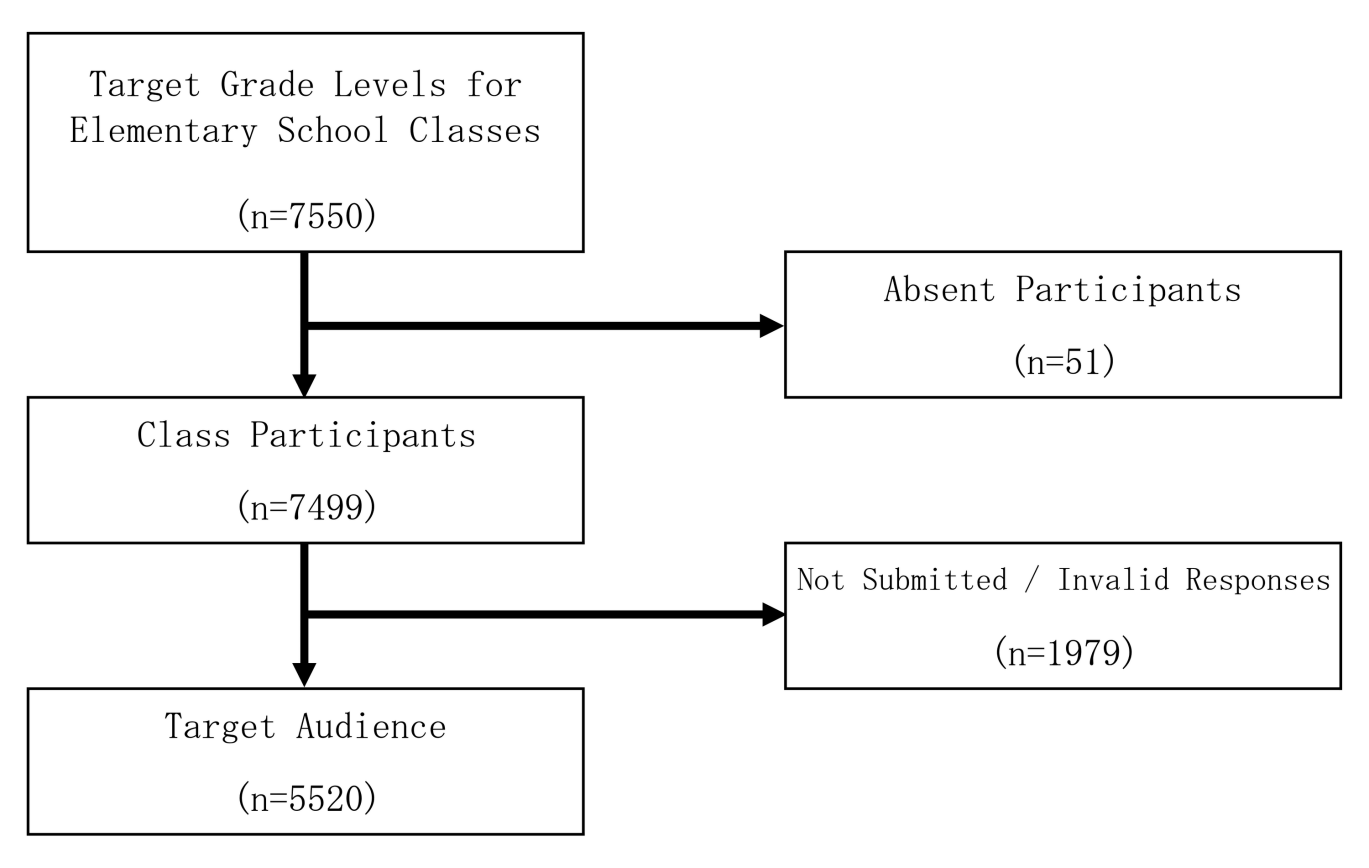
Selection of target participants (elementary school students)

**Figure 2:**
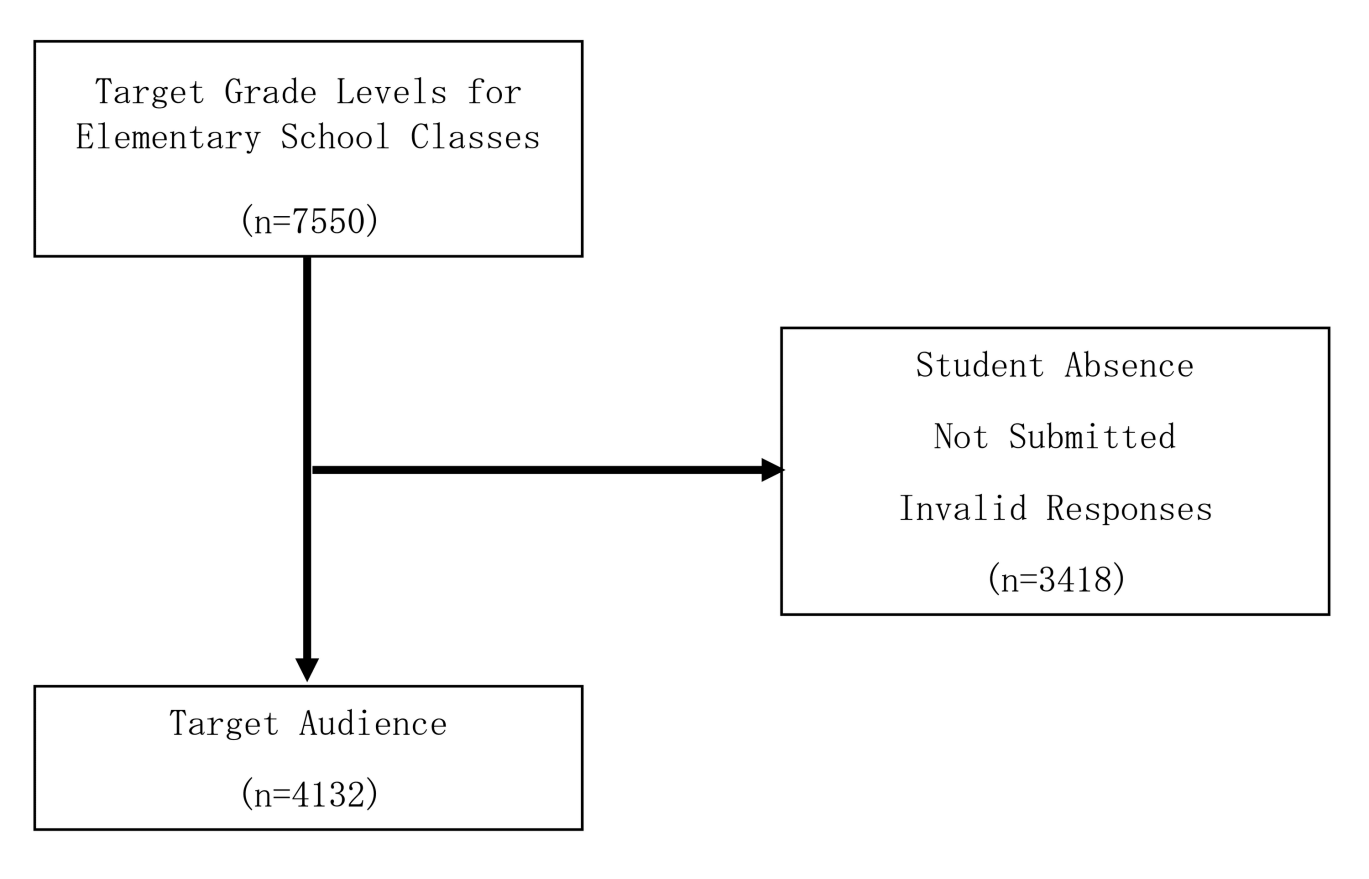
Selection of target participants (guardians)

During the study period, 7,550 elementary school students in the target age group (10 to 12 years) attended classes. After excluding those who were absent on the day of the test, did not submit the test, or provided invalid responses, 5,520 students and 4,132 guardians were included in the study (Figures 1, 2).

Regarding the participants’ characteristics, the age distribution of the elementary school students is summarized in Table 3. Among the students (n = 5,520), 4,324 (78.3%) were 10 years old, 1,151 (20.9%) were 11 years old, and 45 (0.8%) were 12 years old. The age distribution and relationships of the guardians are shown in Table 4. Among the guardians (n = 4,132), 48 (1.2%) were in their 20s, 1,575 (38.1%) in their 30s, 2,370 (57.4%) in their 40s, 102 (2.5%) in their 50s, and 37 (0.9%) in their 60s. With regard to their relationship to the students, 365 (8.8%) were fathers, 3,723 (90.1%) were mothers, and 44 (1.1%) were grandparents.

**Table 3:** Background of the target participants (elementary school students)

Target: Elementary school students			
(n = 5,520)			
		Number	Percentage
Age	10 years old	4,324	78.3
	11 years old	1,151	20.9
	12 years old	45	0.8

**Table 4:** Background of the target participants (guardians)

Target: Guardians			
(n = 4,132)			
		Number	Percentage
Age	In their 20s	48	1.2
	In their 30s	1,575	38.1
	In their 40s	2,370	57.4
	In their 50s	102	2.5
	In their 60s	37	0.9
Relationship	Father	365	8.8
	Mother	3,723	90.1
	Grandparents	44	1.1

The test scores of the students and guardians before and after the class are provided in Table 5. In the nine-point test, the mean score of students significantly increased from 6.3 before the class to 8.7 after the class (difference: 2.39; 95% confidence interval (CI), 2.39-2.41; p < 0.01). Similarly, the mean score of guardians significantly increased from 7.6 to 8.5 (difference: 0.92; 95% CI, 0.91-0.93; p < 0.01).The correct answer rates for each question before and after the class for the elementary school students and their guardians are shown in Table 6. For Question 3, “What are the risk factors for stroke?,” the correct answer rate for “hypertension” significantly increased among the students after the class compared with before the class (68.5% vs. 94.5%; p < 0.001; kappa, 0.08; 95% CI, 0.06-0.10), although the agreement was significantly low. Among the guardians, the correct answer rate decreased slightly after the class, but the difference was not statistically significant (95.5% vs. 94.7%; p = 0.10; kappa, 0.12; 95% CI, 0.07-0.17). For all the other questions, a statistically significant increase in the correct response rate was observed after the class.

**Table 5:** Average test scores of elementary school students and their guardians before and after the class To analyze the test scores before and after the educational intervention, a paired t-test was performed, and the differences and 95% confidence intervals (CIs) were calculated. The significance level for the statistical analyses was set at 0.05 (two-tailed). Confidence intervals are reported with rounding consistent with the precision of the score measurement. P-values are not shown in these tables to avoid redundancy; interpretation of the results is based on differences in scores and changes in proportions. Abbreviations: CI: confidence Interval, SD: standard deviation

	Mean (SD)	Mean (SD)	Difference	95% CI
	Pre-intervention	Post-intervention		
Elementary school students (n = 5,520)	6.3 (2.0)	8.7 (0.8)	2.39	2.39–2.41
Guardians (n = 4,132)	7.6 (1.4)	8.5 (0.9)	0.92	0.91–0.93

**Table 6:** Correct answer rates for each test question before and after the class for the elementary school students and their guardians McNemar’s test was performed for hypothesis testing to compare the correct answer rates for each question before and after the educational intervention and kappa coefficients with 95% CIs were calculated. The significance level for all statistical analyses was set at 0.05 (two-tailed). Confidence intervals are reported with rounding consistent with score measurement precision. P-values are not shown in these tables to avoid redundancy; interpretation of the results is based on differences in scores and changes in proportions. Abbreviations: CI: confidence interval, FAST: Face, Arm, Speech, and Time, Kappa: Kappa coefficient

	Question	Answer	Number of correct answers (%)	Number of correct answers (%)	Kappa	95% CI
			Pre-intervention	Post-intervention		
Elementary school	Symptoms of stroke	One side of the face droops	3,398 (61.6)	5,452 (98.8)	0.03	0.02–0.04
students		Difficulty speaking	4,044 (73.3)	5,440 (98.5)	0.05	0.03–0.06
(n = 5,520)		One side of the body does not move	4,461 (80.8)	5,476 (99.2)	0.04	0.03–0.06
	Response to stroke	Call an ambulance	4,195 (76.0)	5,305 (96.1)	0.13	0.11–0.16
	Risk factors for stroke	Hypertension	3,781 (68.5)	5,218 (94.5)	0.08	0.06–0.10
		Cholesterol	3,596 (65.1)	5,218 (94.5)	0.05	0.03–0.07
		Alcohol consumption	4,017 (72.8)	5,365 (97.2)	0.06	0.04–0.08
		Tobacco	4,249 (77.0)	5,421 (98.2)	0.06	0.04–0.08
	FAST	Face, Arm, Speech	2,972 (53.8)	4,963 (89.9)	0.11	0.10–0.13
Guardians	Symptoms of stroke	One side of the face droops	3,086 (74.7)	3,942 (95.4)	0.14	0.11–0.17
(n =4,132)		Difficulty speaking	3,784 (91.6)	4,028 (97.5)	0.12	0.08–0.17
		One side of body does not move	4,059 (98.2)	4,097 (99.2)	0.03	–0.02 to 0.08
	Response to stroke	Call an ambulance	3,487 (84.4)	3,863 (93.5)	0.26	0.22–0.30
	Risk factors for stroke	Hypertension	3,944 (95.5)	3,914 (94.7)	0.12	0.07–0.17
		Cholesterol	3,501 (84.7)	3,837 (92.9)	0.17	0.13–0.21
		Alcohol consumption	2,688 (65.1)	3,723 (90.1)	0.16	0.13–0.18
		Tobacco	3,132 (75.8)	3,859 (93.4)	0.16	0.13–0.19
	FAST	Face, arm, speech	3,718 (90.0)	3,933 (95.2)	0.12	0.07–0.16

## Discussion

This study examined whether offering stroke classes to elementary school students in a school setting could help them acquire knowledge about stroke symptoms, risk factors, and appropriate responses to stroke onset. Moreover, this study determined whether this knowledge could be transmitted to students’ guardians. After the class conducted by emergency medical technicians, the total test scores and correct answer rates for all questions increased among elementary school students, reflecting short-term improvements in stroke-related knowledge. Guardians also demonstrated increases in total scores and correct answer rates for most questions, indicating corresponding improvements following the educational intervention. Although improvements in knowledge regarding stroke symptoms and appropriate responses may inform future public health education strategies, this study did not directly assess changes in emergency help-seeking behavior, long-term knowledge retention, or stroke-related outcomes. These effects should be examined in future studies using controlled designs and longer follow-up periods. Regarding guardians’ characteristics, the correct answer rate exceeded 90% for all questions after the class. They already had prior knowledge of lifestyle-related diseases, such as hypertension and cholesterol, which are known risk factors for stroke. However, for questions on alcohol consumption and smoking as risk factors, the pre-class correct answer rates were lower than those for the other questions, indicating that their awareness of these risk factors was less pronounced than that of the students. The only question that showed a decrease in correct answer rates after the class was about hypertension. This decline may have occurred because of the difficulty students had in accurately conveying the concept of hypertension to their guardians, leading to a misinterpretation of the question. Furthermore, the kappa coefficients for each question were consistently low, indicating low agreement in responses within the same participants before and after the educational intervention. In other words, response patterns changed following the intervention, which is consistent with the significant changes in correct answer rates for each question identified by the McNemar tests.

While acquiring stroke-related knowledge at a young age may support continued learning and awareness, the findings of this study are limited to short-term changes in knowledge and should not be interpreted as evidence of long-term behavioral change or broader societal impact. Furthermore, the knowledge gained can be valuable if they are bystanders when a family member experiences a stroke [19]. Previous studies have indicated that the presence of bystanders who understand stroke severity and symptoms can reduce delays in the affected patient reaching the hospital and ultimately improve their prognosis [20]. Moreover, knowledge of stroke risk factors can encourage the adoption of a healthier lifestyle from a young age, potentially leading to stroke prevention [14]. This could enhance students’ ability to make appropriate emergency calls during stroke events, potentially reducing the time between stroke onset and hospital admission [21].

This study incorporated a well-known and effective learning style for knowledge retention among elementary school students. We used active learning by integrating conventional lecture-style teaching and encouraging elementary school students to actively participate. The class included audiovisual elements, such as an animated video about stroke, and experiential learning, such as activities aimed at helping elementary school students understand the physical disabilities associated with hemiplegia. In addition, after class, the elementary school students taught their guardians the knowledge they had acquired, thereby reinforcing their understanding. According to the learning pyramid, the average retention rate for lecture-based learning is 5%, but it increases to 10% with reading, 20% with audiovisual materials, 30% with demonstrations, 50% with group discussions, 75% with experiential learning, and up to 90% when teaching others [22]. The learning style adopted in this study included a lecture format and incorporated audiovisual materials and experiential elements. Moreover, by having the students convey the knowledge they gained to their guardians, knowledge retention among the students was likely enhanced.

This study indirectly raised guardians’ awareness via their elementary school children and potentially reached grandparents, who are at a higher risk of stroke [23]. Although there are community activities, in regions such as the state of Michigan in the United States, that raise awareness of the importance of early stroke treatment [24], it remains challenging to target groups that are more likely to witness stroke events, such as older adults. The strength of this study lies in its approach to knowledge transmission from elementary school students to their guardians, providing them with an opportunity to learn. The students may have found it easier to convey the knowledge they acquired in class about stroke using the comic book distributed as supplementary material, in addition to verbal explanations, and the guardians may have been able to better understand the content visually. Involving elementary school students in educating their family members may lead to more effective and widespread public health education within the community [25]. Community education is crucial as CPR training has been shown to double the survival rates in out-of-hospital cardiac arrest cases when the general public undergoes training [26]. Previous studies have reported that elementary school students and many middle and high school students lack knowledge about stroke and require education [27-28]. Furthermore, it is necessary to deepen public understanding of the need for rapid and appropriate responses when stroke is suspected [29]. Continuing this initiative will enable us to educate the broader public about stroke prevention, its symptoms, and appropriate responses to suspected stroke, ultimately reducing delays in bystanders seeking medical attention and improving stroke outcomes. These findings should be interpreted in light of the methodological limitations described below, including the before-and-after study design without a control group and the short follow-up period. Despite these limitations, our findings provide insight into how school-based stroke education may contribute to short-term improvements in stroke-related knowledge beyond the classroom and may help inform the design of future public health education programs.

Limitations

This study has several limitations. First, because a pre-post study design without a control group was employed, the observed improvement in knowledge scores cannot be attributed solely to the educational intervention. Learning effects, familiarity with the test format, and secular trends may also have influenced the results, and causal interpretations should therefore be made with caution. In addition, exposure to other information sources, such as television, newspapers, and the internet, could not be controlled for and may have contributed independently to changes in knowledge.

Second, due to practical constraints, there were minor variations in the timing of the post-class tests among participants. Because the study was conducted at the school and class levels, the timing of post-class assessments was determined by school schedules and events and was not randomized at the individual level. Such variability may have affected knowledge retention and introduced measurement error, thereby reducing score comparability.

Third, the analyses did not account for the hierarchical data structure, with students nested within schools and classes. Although the educational intervention was implemented using a standardized curriculum and unified teaching materials across all schools, ignoring clustering may have led to underestimated standard errors and overestimated precision of the effect estimates.

Fourth, 26.4% of student responses were excluded from the analysis due to absence, non-submission of questionnaires, or invalid responses. These exclusions may have introduced selection bias and limited the generalizability of the findings. Because individual-level demographic information and baseline data were not available for excluded participants, direct comparisons with included participants could not be performed. In addition, in households with multiple participating children, the possibility that the same guardian completed the questionnaire more than once could not be completely ruled out, which may have introduced measurement bias.

Fifth, this study was conducted in a single municipality in Japan (Akashi City, Hyogo Prefecture), which may limit the generalizability of the findings to other regions with different educational systems or emergency medical infrastructures. Furthermore, we were unable to distinguish specific pathways of knowledge transfer to guardians, such as direct explanations by children versus learning through supplementary materials (e.g., comics).

Finally, post-class assessments were conducted within a short period after the educational intervention; therefore, long-term knowledge retention and behavioral change could not be evaluated.

## Conclusions

In this study, short-term improvements in stroke-related knowledge were observed among elementary school students following a school-based stroke education program conducted by emergency medical technicians, with similar improvements observed among guardians. These findings reflect immediate, group-level changes in knowledge scores and suggest the potential for intergenerational knowledge sharing. However, this study did not directly assess behavioral change, long-term knowledge retention, or community-level stroke outcomes. Future studies using controlled designs and longer follow-up periods are needed to determine whether these short-term knowledge gains translate into sustained behavioral change or improved clinical outcomes.
